# An Exploratory Study of Extreme Sport Athletes’ Nature Interactions: From Well-Being to Pro-environmental Behavior

**DOI:** 10.3389/fpsyg.2019.01233

**Published:** 2019-05-28

**Authors:** Tadhg Eoghan MacIntyre, Andree M. Walkin, Juergen Beckmann, Giovanna Calogiuri, Susan Gritzka, Greig Oliver, Aoife A. Donnelly, Giles Warrington

**Affiliations:** ^1^Health Research Institute, University of Limerick, Limerick, Ireland; ^2^Department of Physical Education and Sport Sciences, Faculty of Education and Health Sciences, University of Limerick, Limerick, Ireland; ^3^Faculty of Sport and Health Sciences, Technische Universität München, Munich, Germany; ^4^Department of Public Health and Sport Sciences, Inland Norway University of Applied Sciences, Elverum, Norway; ^5^Department of Psychology, Dresden University of Technology, Dresden, Germany; ^6^Dublin Institute of Technology, Dublin, Ireland

**Keywords:** nature connectedness, extreme sport, well-being, resilience, green exercise, blue exercise, restorative space, emotion – mood

## Abstract

Traditionally, perceptions about extreme sport athletes being disconnected from nature and a risk-taking population have permeated the research literature. Drawing upon theoretical perspectives from environmental, sport, organizational and positive psychology, this qualitative study attempts to explore the lived experiences of four male and four female extreme sport athletes. The purpose of this study was to gain insight and understanding into the individuals’ attitudes toward the benefits of extreme sport activities for well-being, resilience and pro-environmental behavior. Eight participants (Mean age = 40.5 years; *SD* = ± 12.9) provided written informed consent to partake in semi-structured interviews. Each athlete provided written consented to allow the publication of their identifiable data and in order to facilitate sharing of their autobiographical account of their experiences. After conducting thematic analysis, meta-themes that emerged from the analyses were as follows: (a) early childhood experiences, (b) the challenge of the outdoors, (c) their emotional response to nature, (d) nature for coping, (e) restorative spaces, and (f) environmental concern. The findings convey great commonalities across the participants with regard to their mindset, their emotional well-being as well as their connectivity with nature and attitudes toward the natural environment. The cognitive-affective-social-behavioral linkage of the benefits of extreme sport participation for well-being, psychological recovery and pro-environmental behavior are highlighted. This study examining the lived experiences of extreme sportspeople provides a novel contribution to our contemporary understanding of extreme athletes’ relationship to nature and its commensurate impact upon well-being and pro-environmental attitudes. The findings suggest that extreme sport participation, while inherently risky has psychological benefits ranging from evoking positive emotions, developing resilience and life coping skills to cultivating strong affinity to and connection with nature and the natural environment.

## Introduction

It has been widely assumed that extreme sports people are for the most part risk taking, sensation seeking individuals who lack connection to the natural world (Zuckerman and Neeb, 1979; Breivik, 1996; Zuckerman, 2009). However recent research shows that while this is the case for some high-risk sport participants other additional behavioral and motivational benefits may be derived from the exhilaration of the extreme sport challenge. Preliminary evidence suggests that in some extreme sports emotional experiences of nature constitute a compelling component of the overall sporting experience. For instance, interviews with extreme sport participants suggested that they cultivate feelings of connection to the natural world (Brymer and Gray, 2010). Extreme sports are considered not simply as outdoor leisure activities where the most likely outcome of mismanaged mistake is accident or death but the experience of approaching danger is integral to these sports (Brymer and Oades, 2009). This paper attempts to reconcile the commonly accepted assumption of the egocentric nature of extreme sports with the lived experiences of individuals across a range of extreme sports. It builds upon research by Willig (2008) which discusses the positive human experiences that extreme sport participation can have on an individual and also how it can enhance their relationship and connectedness with nature and the natural environment (Brymer and Gray, 2009, 2010). We are concerned with how human-nature interactions benefit an individuals’ well-being and coping, in addition, to their nature connectedness.

### Psychological Benefits Positive Emotions and Psychological Recovery

Research suggests that participation in extreme sport activity might develop valuable personal attributes such as courage and humility (Brymer and Oades, 2009). Other research suggests high risk sports allow participants to explore and embrace “fundamental human values” which can have formative and “transformational benefits” (Brymer and Schweitzer, 2013).

Exposure to nature has been shown to provide a range of psychological benefits with a recent review of high-quality systematic reviews showing strong evidence for improved affect as a consequence of nature contact (van den Bosch and Ode Sang, 2017). The interaction of an extreme sport athlete with natural environments might provide important experiential drives as well as help the athlete cope in moments of high stress. Anecdotal evidence from outdoor recreation enthusiasts suggests that nature can provide powerful emotional experiences, which can draw people to engage in activities such as hiking, climbing, and trekking. Some philosophical accounts have also emphasized how being in contact with nature, away from urbanization, can provide strong emotional reactions leading to a deep commitment toward nature (Gelter, 2000). Although the literature on nature experiences in extreme sports is still scarce, both the extant quantitative and qualitative literature shows how emotional attachment to the natural world as well as expected psychological benefits of being in contact with nature are central to the experience of outdoor recreation and nature-based exercise (Anderson et al., 2009; Calogiuri, 2016; Flowers et al., 2016; Hervik and Skille, 2016; Calogiuri and Elliott, 2017).

Theoretical explanations, such as Kaplan’s attention-restoration theory (ART; Kaplan and Kaplan, 1989) suggests a cognitive benefit of nature contact, which has been even applied in the contests of exercise or physical activity (Hartig et al., 1991, 2003; Bodin and Hartig, 2003; Aspinall et al., 2015; Calogiuri et al., 2015; Calogiuri et al., 2018). A core assumption of ART is that *directed attention*, the act of focusing on information of no spontaneous interest by inhibiting competing stimuli (e.g., concentrating in repetitive or cognitively demanding tasks), is a limited cognitive resource that can be exhausted leading to mental fatigue. According to ART, different environments can elicit psychological restoration depending on four key qualities of the person-environment relationship: *fascination* (the extent to which the environment triggers spontaneous and effortless attention), *being away* (the extent to which the environment provide an opportunity to take distance from tasks, hassles, daily routines, etc.), *coherence* (the extent to which an environment is perceived as coherently ordered, non-chaotic), and *compatibility* (the extent to which an environment meets the inclinations of the person at the time). Natural environments are often characterized by features that are particularly efficient in eliciting psychological restoration avoiding excessive excitement, a process referred to as soft-fascination. A complimentary theory, *stress reduction theory* (SRT; Ulrich, 1984; Ulrich et al., 1991), grounded in the concept of “stress recovery” or “restoration,” is based upon the premise that restoration or recovery from stress involves numerous *positive* changes in psychological states. For example, changes in emotional states including reduced levels of negative affect would be predicted to reduce stress.

Prior research has been largely grounded in the aforementioned theoretical accounts from environmental psychology which have been concerned with attention restoration and stress recovery, which are a narrow set of outcomes given the corpus of evidence on the psychological benefits of human-nature interactions (Frumkin et al., 2017; van den Bosch et al., 2017). Recently researchers have applied other perspectives including dynamical systems approaches (Immonen et al., 2018). Organizational psychology offers new insights regarding the concept of psychological recovery which are integrated with the construct of resilience and provide additional testable hypotheses. Recovery, as Sonnentag et al. (2017) posit, refers to the process of psychological unwinding recuperating or restoring physical or mental resource that counteracts the stress process triggered by job demands and other stressors. Research on recovery processes has centered mainly on psychological detachment in work-related circumstances with findings typically demonstrating a decrease in exhaustion and an increase in perceived resources. This perspective, based on psychological resources theory (Hobfoll, 1989), can illuminate our understanding of nature as a restorative place and augment the literature on therapeutic landscapes (Bell et al., 2018).

### Resilience and Extreme Sport

Recent research has discussed how high-risk sports participants develop a positive and distinctive relationship with nature and their surrounding environment which in turn can benefit the individual (Brymer et al., 2009; Brymer and Gray, 2009, 2010). The challenge of the extreme sport environment may be the process that stimulates this positive human nature interaction through the process of resilience. The lens of the resilience construct (see Bryan et al., 2017) has not been readily applied to understanding human-nature interactions. Exploring resilience as an outcome of the positive and adaptive response to the challenge of extreme sports in nature offers an opportunity to explore how stress, psychological resources, coping and psychological growth interact.

The concept of connectedness with nature or the similar concept of nature relatedness, is defined as the extent to which an individual experience being emotionally connected to the natural world (Mayer and Frantz, 2004; Nisbet et al., 2009). This concept has been proposed as having relevance not only for the extent to which a person conceive and experience his or her relation with the natural environment, but also for his or her mental health and well-being. A meta-analysis by Capaldi et al. (2014) showed that individuals who are more connected to nature tend to experience more positive affect, vitality, and life satisfaction compared to those less connected to nature. Associations have been found also for connectedness with nature with personality traits like agreeableness and openness (Nisbet et al., 2009). Moreover, individuals with more positive attitudes toward nature were also found to spend more time in natural environments (Beery, 2013; Calogiuri, 2016; Flowers et al., 2016), increasing the opportunity to engage in favorable restorative experiences, reducing and preventing stress.

The frequency with which people interact with nature as a child appear to be particularly crucial in people’s development of feelings of connectedness with nature. Studies based on self-reported recall of childhood experiences of nature have found that frequent nature-interactions as a child are associated with increased emotional attachment to nature as well as more frequent nature-based activities in adult life (Asah et al., 2012; Calogiuri, 2016). There is also evidence indicating that attitudes toward as well as the subjective experience of nature-interactions can be strongly influenced by the frequency of childhood experiences in nature (Ward-Thompson et al., 2008).

### Pro-environmental Behavior and Environmental Concern

The topic of environmental sustainability has been emerging as a major social issue in the present century not only in environmental sciences but also in public health. Warnings about the risks posed by environmental issues, such as air, water, and soil pollution, co-hazards of noise and air pollution, climate change and ultraviolet radiation have been raised at the highest levels including UN and EU (e.g., UN SDG’s; Science for Environment Policy, 2016). In addition to our connectivity with nature, place attachment (an emotional, cognitive, and functional bond with a place) has emerged as a key psychological component fostering sustainable behavior (Restall and Conrad, 2015; Geng et al., 2015). Studies have found significant associations of both our relationship with nature (e.g., Mayer and Frantz, 2004; Nisbet et al., 2009) and place attachment (Halpenny, 2010; Scannell and Gifford, 2010) with environmental concerns and commitment to pro-environmental behaviors.

The overall aim of this study is to explore the lived experiences of extreme sports participants through semi-structured interviews. Lived experiences of extreme sport participants has been previously reported using the lens of environmental psychology (Brymer, 2010; Brymer and Schweitzer, 2015). This exploratory study will seek to add an additional layer of evidence from across a myriad of extreme sport activities including mountaineering, ski-flying, ultra-endurance open-water swimming, white-water kayaking and big wave surfing and apply the lens of positive psychology frameworks including the constructs of psychological recovery and resilience.

## Materials and Methods

### Participants

Eight adults who engaged in extreme sport activities (See Table 1) volunteered to participate in the study (4 males and 4 females; Mean age = 40.5; *SD* = 12.9). Inclusion criteria were that all participants had to be over 18 years of age, were currently or had previously participated in extreme sport activities. The pre-selected sample were contacted by email initially and waived the right to anonymity which was part of the institutional ethical approval (EHS Ethical Approval No. 2016 _11_20 EHS).

**Table 1:** Participant name, gender, nationality and sporting experience.

Name	Gender	Nationality	Sporting experience
Easkey Britton	F	Irish	Former international professional surfer and big wave surfer
Chris Bryan^∗^	M	Irish	International competitor in long distance open-water swimming.
Rosie Foley^∗^	F	Irish	Channel swimmer and former Ireland rugby international (36 caps)
Sandra Hyslop	F	British	White-water kayak competitor
Jim Kennedy	M	Irish	Ultra-endurance kayak competitor
Andreas Küttel	M	Swiss	Three time Olympian in ski-jumping and ski-flying competitor
Tehillah McGuinness^∗^	F	South African	International professional surfer and big wave surfer
Humphrey Murphy	M	Irish	Everest mountaineer and white-water kayaker

^∗^*denotes active competitor*.

### Materials

A semi-structured interview guide was pilot-tested and developed based on a deductive approach designed to test theories of human-nature interactions (see Table 2).

**Table 2:** Interview guide structure.

Interview section	Aim	Exemplar questions
I. Introduction	To explain the focus of the study and address any initial questions in advance of the discourse.	Do you have any questions before we commence?
II. Rapport Building	To develop trust with the participant by referring back to their prior experiences.	Please tell me about your career achievements and personal milestones.
III. Identification of Adversity	To examine coping strategies, experience of post-traumatic growth and coping with daily hassles.	What adversities have you faced in your sporting career and how have you coped and thrived in response?
IV. Green and Blue Exercise Participation	To explore frequency, type (individual/group), intensity of exercise.	What activities do you do outdoors?
V. Place Attachment	To explore place blindness, emotions associated with different natural spaces.	Do you have a favorite natural space?
VI. Access to Nature	To address barriers to engaging with nature.	Are there any other barriers or risks to being active in nature?
VII. Environmental Sustainability	To explore attitudes toward the environment and sustainability.	What are your views on sustainability and the environment?
VIII. Technological Nature	To probe if they augment authentic nature with tech. nature.	Do you need to be in nature for it to impact upon you?
IX. Additional comments	To provide an opportunity to discuss any other issues that they would like to raise.	Anything else you would like to add on how blue or green natural spaces can benefit health
X. V. Closure	To ensure any anxieties or concerns are addressed before ending the interview.	The next step will be approval of the transcript.

### Procedure

After providing written informed consent to participate in the study, participants were invited to interview in a place convenient to them for a face-to-face interview (*N* = 2) or to alternatively, conduct the interview online (*N* = 6). Each athlete also provided written consent to allow the publication of their identifiable data in order to facilitate sharing of an autobiographical account of their experiences. Interviews were all conducted in English and the duration ranged from 40 to 60 min with a resulting average word count of 5,000 words (Mean = 5,390 words). Interviews were audio recorded and full transcripts were created and subsequently approved by each participant.

### Analysis

The raw data quotes from the transcripts were thematically coded (see Supplementary Materials) based on procedures for qualitative research (Robson and McCartan, 2016). An *a priori* coding frame coupled with a theory driven approach to the development of the semi-structured interview guide facilitated an efficient coding process. The second author immersed themselves in the data by reading all transcripts a number of times. Next, they conducted an inductive hierarchical content analysis to convert raw data quotes into sub-themes under the previously identified meta-themes. Verification procedures included independent coding of the transcripts (inter-rater reliability: 85%). A third researcher provided oversight and took the role of critical friend in the coding process and re-coding process.

## Results and Discussion

### Qualitative Analysis

The meta-themes that emerged from the analyses were as follows: meta-themes that emerged from the analyses were as follows: (a) early childhood experiences, (b) the challenge of the outdoors, (c) their emotional response to nature, (d) nature for coping, (e) restorative spaces, and (f) environmental concern.

#### Early Childhood Experiences

**Figure 1:**
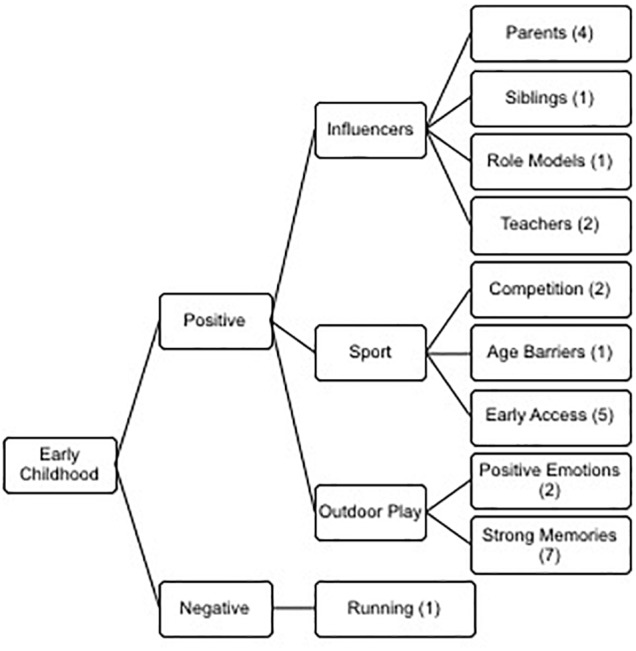
Thematic analysis of the meta-theme early childhood displaying the four themes and nine sub-themes.

The strong influence of childhood engagement in nature has been noted previously (Ward-Thompson et al., 2008; Asah et al., 2012; Calogiuri, 2016). In our analysis of the interviews two major themes emerged under this major theme (See Figure 1): Positive (sub-themes: outdoor play, sport and influencers) and negative (sub-themes: running). Our former ski-flying competitor, Andreas Küttel, who had five FIS World Cup wins between 2005 and 2007, reflected on the importance of his early childhood experiences in the outdoors and how he is now trying to ensure that his son has similar opportunities:

My best childhood memories are just being in the forest or somewhere like the beach so of course I try to repeat that with my son also …going on holiday camping are some of the best childhood memories of course. Being in the forest, making fire, swimming in the lake, hiking…the nature in Switzerland where I grew up is fantastic.

He recalls that: We could just go to a riverbank and stay the whole day there…we would make the dams and jump on the stones… kids are sort of happiest when they can just take natural things.” Jim Kennedy describes the propensity for outdoor physical activity growing up by the river Lee in Cork city:

It was pre-TV if your heads can imagine that. There were maybe 20 to 30 kids in the streets so outside for us was just the natural thing, we didn’t know any better, we would run everywhere.

Interestingly, Sandra Hyslop, a fellow kayaker states that she hated running- “It’s just really uncomfortable for me to do” conveying the multiple trajectories for individuals engaging in outdoor play and physical activity during their formative years.

Andreas described his progression from an early age directly into the sport ski-jumping: *“I have some pictures when I could hardly walk and I was already on skis…. That was in summer you know- summer ski jumping on plastic hills.”*

He was the youngest in his club that was making trips to St. Moritz for training:

It was exciting as an eight to nine year old to travel with teenagers and stay overnight. You get all the skills like pack your own bag and being independent at a pretty early age.

Sandra Hyslop, who won the Adidas Sickline extreme kayak race (e.g., Kayak race on category Class 5 white-water on river Oetz), noted both the negative and positive contribution of early childhood experience: *“We always used to go hill-walking as a family and dad was really into caving which we didn’t enjoy quite so much but that was the weekend thing as a family.”*

She continued “I just always like water…I can’t remember not being able to swim.” In England, she recalled that she wanted to join the swim club at 6 years of age but they refused as they didn’t take people until they were 8 years old. A major life event was to provide an opportunity for Sandra:

We moved to America when I was seven and they are pretty big on swimming in the States [United States] so I joined the swim club there so I started and used to go like every day in the Summer… so it was probably pretty serious swim training from about age eight and I guess and that probably built the fitness and confidence in the water.

Similarly, for Irish Surfer Easkey Britton, 8 years of age was recalled as a key milestone for her:

I’m a lifelong surfer and I’ve been competing since the age of eight and have surfed professionally, it’s something that is a huge part of my life. From an early age surfing and the sea has been a huge part of who I am and this has continued to influence me daily.

Fellow surfer Tehillah McGuinness sums up her early introduction concisely: “Surfing chose me, I didn’t choose surfing.” Big wave surfing differs from other competition events in that participants engage in tow-in-surfing and thus the waves are typically of a greater magnitude. However, she described that at 8 years old, prior to surfing, she got into long distance running: “that was like my dream to be in the Olympics.” Rosie Foley describes how as a child she would try any sport with Anthony and her younger sister Orla: “Any sport that was on the TV, we were doing in the front lawn.”

The participants described early access to the outdoors as a gateway into sport. Andreas specialized early for ski-jumping (e.g., progressing to Ski-Flying) and others engaged in more of a multi-sport approach but typically were in outdoor sports by approximately 8 years of age. At this age, adult support in addition to peer learning is often required.

Parental influence was instrumental for Andreas Küttel who recalled that:

My dad was a physical coach for the ski jumping team so I was very in touch with it and the ski jumping center is in my home town.

This provided him with an opportunity to access the ski-jumps, observe both peers and expert performance, receive coaching, all with the support of his father. Sibling support was noted as influential for Tehillah McGuinness who noted:

I think I was quite a Tom-Boy – I mean growing up we were all incredibly close – my mum kind of had us in pairs – so my brother and I when we were younger, we were a year apart so I would do everything with him and his friends.

Interestingly, Rosie Foley, who had grown up in the midst of a patriarchal rural Irish society, highlighted those influencers who had overcome social barriers to facilitate early childhood opportunities for participation regardless of gender. She recalled how a local swim teacher had introduced her to swimming in the river Shannon:

Peter Lacey was a gentleman renowned in this area and he used to teach people in the Pier Head how to swim with a kind of a homemade leash structure that he’d hold onto people [they were tethered] and that’s how he got them going.

In swimming, the inspirational role of coaches was also noted by Chris Bryan:

The coach who was at Ennis Swimming Club, Sean O Sullivan, was the father of my best friend. I regularly stayed with them at Spanish Point [coastal town 35 km from Ennis, Co.Clare]. He would have been a major influence. You could see the passion they had that really then helped me, kind of, glue to the open water swimming scene.

Britton found her influence in a fellow female sportsperson who inspired her to take her sport to the next level:

Sarah Gerhardt, she is another example of someone who has inner passion for the sea and surfing with her science and she’s a lecturer based in California. But she surfs outside near Santa Cruz and was one of the first women …to pioneer one of the big wave spots called Mavericks in the 90’s … so this was the first time we would have seen a woman in a wetsuit in cold water, all the things that make up who I am and women who surf in Ireland but have never been exposed to, seeing that that’s possible in the world and where you can go with it.

#### The Challenge of the Outdoors

**Figure 2:**
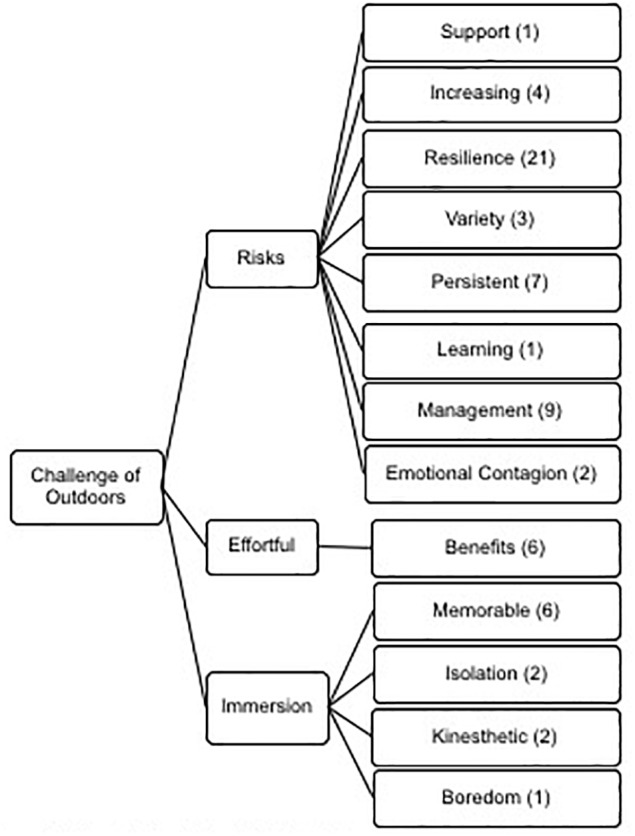
Thematic analysis of the meta-theme challenge of outdoors displaying the three themes and sub-themes.

Extreme sport and ultra-endurance activity range in their threat to individual survival. For example, the Devizes to Westminster kayak race is renowned as a rite of passage for member of the elite military forces and while an ultra-endurance event, the 77 portages, 125 miles of both river and canal (much of it in darkness) poses risks for athletes’ welfare and physical well-being. Other examples, like round Ireland kayaking, first descents, Channel swims, extreme kayaking competitions and summiting Mount Everest on first glance would suggest that they are all extreme in nature, but our participants’ voices articulate most clearly how the myriad of feats posed differential risks depending on context and circumstance (see Figure 2). Three major themes emerged under this meta theme in our analysis of the interviews: Risks, Effortful and Immersion.

Frühauf et al. (2017) found that challenge in the extreme sport of freeriding for some participants provided an opportunity to explore and stretch their personal limits. Extreme sports create an opportunity for participants to self-determine their own level of challenge. This theme resonated with Easkey Britton who said:

You’re always on the edge of challenge because of the environment and definitely big wave surfing you’re in extreme situations all the time so plenty of adversity in that sense…there is a lot of fear as well as attraction as we are drawn to water environments.

Humphrey Murphy, one of approximately 60 Irish people who have successfully climbed Mount Everest noted:

Everest was a relatively straight forward one of all of them. The Everest trip in a way there was something artificial about that because of the level of structure there is around it and in terms of signature trips.

In contrast, his expedition kayaking trips were much more challenging environments.

Caucasus and Siberia and they were again going out to do rivers that were very challenging but again very remote in so far as you step into gorges for a week at a time and really didn’t have much of an option than to stay in them and kayak down them.

Kayaker Sandra recounts how coupling the physical and technical challenges is the toughest aspect for her, so competition scenarios create an added pressure:

What’s nice is the challenge. You’ve got to be on the athletic edge physically pushing as hard as you can and still have enough to nail the technical aspects. I did the white-water grand prix one year that’s definitely the most scared I’ve been ever been racing.

Stress can be ameliorated by taking on challenges in new ways as Sandra explains:

When I started kayaking we used to love just swimming down the rapids … if you’ll happily swim down it then why wouldn’t you kayak it- the worst that can happen is that you’ll swim and so I think that’s why being a strong swimmer is huge for your white-water confidence.

While swimming in white-water has its’ own challenges, open-water swimming on the other hand provides a unique environment as Channel swimmer Rosie Foley recalls: “I was reared in the water in Killaloe so I don’t mind the blackness and that darkness.” Rosie’s extreme feat was the Channel swim (22 miles at its narrowest point) in a time of 15 h. 53 mins.

#### Their Emotional Response to Nature

**Figure 3:**
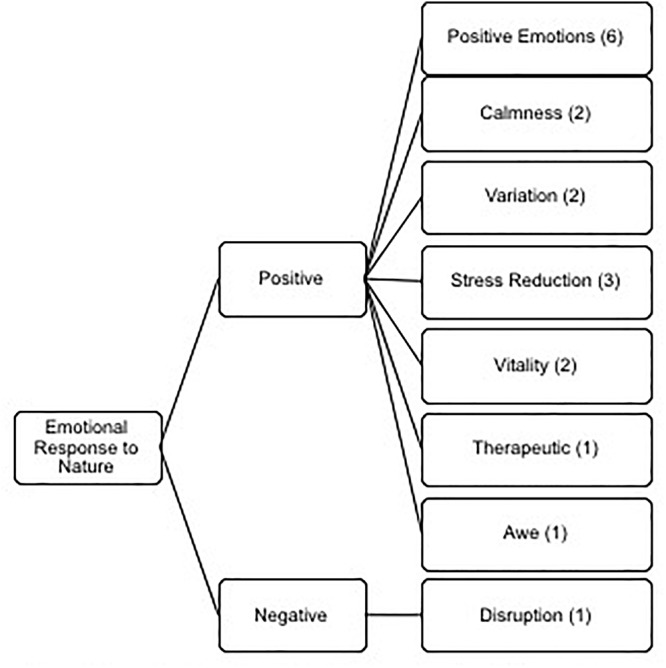
Thematic analysis of the meta-theme emotional response to nature displaying the two themes and eight sub-themes.

Our participants emotions from interacting with nature were predominantly positive with only one potential negative outcome. The resulting themes were (See Figure 3) positive (sub-themes: positive emotions, calmness, variation, stress reduction, vitality, therapeutic and awe) and negative (sub-theme: disruption).

Firstly, Sandra extols the virtues of being on the water: “Pretty much whatever the river it’s like once you’re in the boat I’m pretty much always happy.” She reflects that “just being on the water is awesome... I guess how or are you out to relax and enjoy yourself.” Activity in nature as a source of positive emotions aligns with the recent scientific literature (Lumber et al., 2017).

Andreas described how nature is integral to his daily life and played a role for him when he was a competitor:

It’s just amazing being in the nature. So yeah, it gives me absolutely a lot of energy on a daily basis but also for special occasions it gives you calmness. An important part of my competition preparations was being out in nature.

In contrast to this calmness referred to above, Jim is inspired by the inherent vitality of nature:

Where the sea meets the land, is probably the most powerful place in the world. That line. That coastal line where the white waves, that’s probably the most, besides volcanoes, or earthquakes, that’s probably the most incredibly natural forceful place in the world, a powerful place in the world. It’s an amazing area.

The contrasting experiences recounted by our participants convey how nature offers a myriad of experiences with different emotional outcomes as Sandra explains:

It kind of depends on the river, which offers like different rewards I guess, you’re getting the adrenaline rush if you are on the harder stuff and kind of that sense of achievement when you make it through the rapid.

For climber and kayaker Humphrey geography is no barrier:

When I am in a particular environment be it the sea or a mountain environment or an air sport environment...it evokes certain reactions within me regardless of whether that’s in Ireland Kerry or the Himalayas.

He explains how we can disrupt the positive emotional response in certain situations:

Green spaces and blue spaces are unquestionably therapeutic whether we want them to be or not. However, if we do a sort of boot camp approach to the outdoors, if we are shouting at people, to do more trying, to get them to go further or faster and so on, we actually we undermine the meanings of those environments and we create a negative environment.

#### Nature for Coping

**Figure 4:**
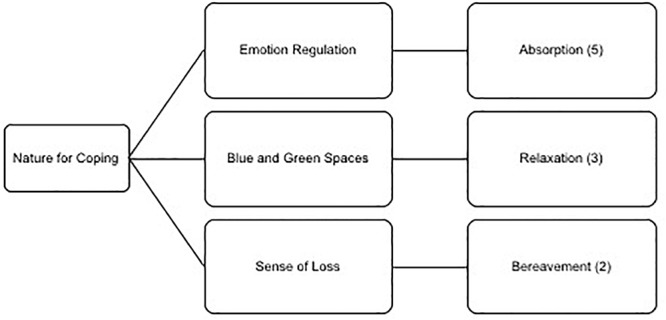
Thematic analysis of the meta-theme nature for coping displaying the three themes and three sub-themes.

Nature has long been established as providing a role in coping, both in human geography (e.g., therapeutic landscapes, Bell et al., 2018) and in explanatory accounts of human-nature interactions (e.g., stress reduction theory, Ulrich et al., 1991) and within the literature on psychological recovery (Sonnentag et al., 2017). Three themes emerged, see Figure 4, from our analyses: Emotional regulation (sub-themes: absorption, competition) blue and green spaces (sub-theme: relaxation) and sense of loss (sub-themes: bereavement, injury). Perception of awe has unique beneficial effects for mood, according to a recent review by Lumber et al. (2017).

Our participants similarly conveyed this idea of emotional regulation through nature expressed by open-water swimmer Chris Bryan: “The beach in Sri Lanka. It has really, really strong currents. I really enjoy the rough seas as well. I swam there every day last year. It humbles me.” The sense of flow, which has been evident in prior research (Brymer and Gray, 2009), appears to be linked to the multi-modal memories of those experiences as described by surfer Easkey Britton:

Obviously the emotional connection has to be there. I think it can be a very powerful especially memory. I think memory gets formed or shaped in water from experiences with being immersed in water. As a surfer you can recall exactly, a wave only lasts a few seconds but you can recall that exact moment and it could be years and years ago whereas you can’t really remember so clearly what you did yesterday.

Competitive experiences provide a stimulus for a change in mindset. Chris Bryan recalled that “as I have grown it kind of had to transform, every time I had to travel internationally there was a different experience, a different venue.”

Natural spaces, both blue and green, aided stress reduction and promoted a relaxation response. Two participants explain this process. Firstly, Everest climber Humphrey Murphy states that “I can physically relax in seconds, that’s what I can do when I get into nature. I just let my mind go.” For Rosie Foley “the emotions are just pure relaxation and just that lovely feeling of this is this is where I’m supposed to be. This relaxes me this gets everything out of me.” This cleansing metaphor highlights the de-stressing effect of human-nature interactions.

One clear example of this is how Rosie, who’s brother Anthony Foley (1973–2016) passed away in 2016, coped with the bereavement:

When I’m swimming I can cry, when I’m swimming I can do whatever the hell I want, when I’m swimming and it’s me on my own and its fine and I’m not upsetting anyone else …that’s how it helps me cope.

#### Restorative Spaces

**Figure 5:**
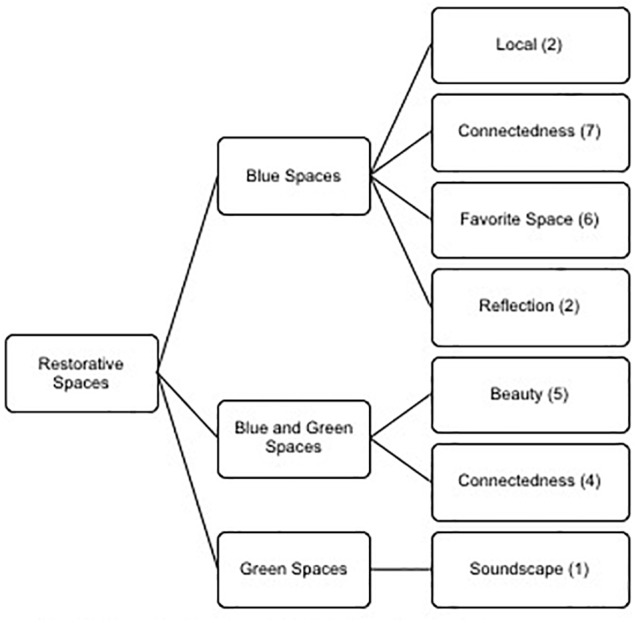
Thematic analysis of the meta-theme restorative spaces displaying the three themes and seven sub-themes.

Three major themes emerged under this topic in our analysis of the interviews (See Figure 5): Blue Spaces (sub-themes: imaginary, local, connectedness, reflections, favorite natural space), Blue and Green Spaces (sub-themes: beauty and connectedness) and finally, Green Spaces (sub-themes: soundscape). Blue natural spaces are different to green, with potentially greater salutogenic effects, and discrete pathways (Gascon et al., 2017).

Not surprisingly, given that more than two-thirds of our participants had extreme sport experiences in water sports, their preference for blue natural spaces were typically higher among our participants. Rugby international, and subsequently a Channel swimmer, Rosie Foley said:

I’m more blue, I think, I’m more blue but it’s funny like I’m drawn to places where there is water…Lough Derg to me would equate in a sense to the channel and I always felt that it was on my own backyard this is what I’ve spent so much time on the lake I love it here.

Rosie lives adjacent to the Lough Derg Blueway (e.g., a series of bespoke multi-recreational trails on Ireland’s third largest lake over 24 miles in length totaling 50 square miles) and thus the connection to water is not unexpected. Similarly, Jim Kennedy has access to a body of water on his doorstep:

Where we kayak we have a premises there, a site, and you take four steps from it and you’re in the sea, it’s on the sea, on a tiny little slip about 25–30 feet high so most beautiful, spectacular place for what we do.

His traveling adventures in Mexico evoke a unique sense of connectedness across the ocean: “Mexico is our second home at this stage, so when people are on, my friends are on the water, I’m on the water here, we’re actually touching each other, in a kind of a strange way. It’s the same water.” Sensation seeking theories (Zuckerman, 2009) fail to account for the banal beauty of the varied experiences of our performers. As Sandra states:

I think I guess some people in the white-water community find me a bit strange cause I am perfectly happy paddling on flat water for an hour and it’s not as good probably as the white water but … I just like being on the water and I can enjoy great flatwater.

This quote highlights that it’s neither the challenge nor the competition that defines the experience but the engagement and absorption with the activity in nature. This connectedness with nature is conveyed by a lucid description from Humphrey Murphy: “the outdoors is a more real place than most else in my life. This idea resonates with Andreas who explains “I often tried to find a place where I feel this is like a center of energy.” Open-water swimmer Chris explains:

I used to go out to Killaloe quite a lot…And again it was that combination of cold water and just, eh getting away from it all, that it was actually the one time that I could stop and frame my thoughts. So that was actually really valuable to me. And sometimes I didn’t appreciate the value that had, in that I didn’t get out there enough to do that.

Sandra echoes these sentiments: “I love getting out in nature, if you are by yourself enjoying some space and getting away from everything.” Experiences in nature appear to have a restorative function –they provide energy to the participant and instill a sense of recovery. The role of nature in promoting psychological recovery through positive emotions (Sonnentag et al., 2017) resonates through the participants descriptions and merits further investigation.

This restorative function of nature was mirrored in descriptions of their favorite places. As Easkey explained: “I travel a lot and coming home has always had the restorative aspect and back home to Donegal where I grew up and that has that kind of energy.” In a similar vein Andreas describes his hometown:

There is a lake and we had a small place at this lake. When I was an athlete I was actually swimming there before breakfast. My wife would not call it swimming as I was just making 100 m out and in again. But I was in the water practically every morning in Summer, cycling home, having breakfast, and starting training at nine. And then after the training at four or five o’clock I went again to this place, doing stretching, and it was all so organic…I guess today, you would call it “exercising mindfulness.”

Jim’s favorite place is idyllic but imaginary:

I have never actually physically seen it. But it’s a tiny little beach, it’s about 50 feet wide, and there’s shells on the left-hand side and there’s a slope on the beach, and I know it intimately.

There is a cognitive flexibility in the approach of our participants in how they access nature and optimize their interactions as described by surfer Easkey Britton in conveying how she goes to green natural spaces for well-being:

If I go to say London for work within a few hours or a few days I feel like claustrophobic and I need to be near the sea –nature yes definitely beautiful forests, yes, I love the forests but the sea that’s kind of what mentally does it for me.”

Aesthetics of nature resonate clearly with our participants, supporting the findings of Lumber et al. (2017). As the quote from Tehillah stated:

When we moved from Jeffreys Bay to the United Kingdom, as I said we moved from living right – basically I stood up in bed and opened my window and I could see the sea… And we moved from that to a little village where you wake up and see sheep in the field, it was green and it was as beautiful as that was it was different and for me, it kind of evokes different emotions.

The ubiquitous influence of nature, whether blue or green, is integral to Easkey’s connection with nature: “I’m also passionate about overcoming that sense of separation and disconnect I suppose we have between land and sea nature and culture and ourselves and nature.” For Rosie “the green has brought us some great places around the world I have to say so if we are walking the dogs or walking with the kids or we are going cycling.” Soundscape is also important in this restorative function of nature as Andreas explains:

I’m having a workshop with the Swiss ski-jumping team and what we actually do, sometimes out in the nature, is just lay down, breath and say what do you hear and suddenly you hear all kinds of birds and I hear all kinds of noises.

What is termed *soundscape* (Aletta et al., 2016), which concerns the sounds perceived and understood by individuals, is integral to the multisensory experience of nature.

#### Environmental Concern

**Figure 6:**
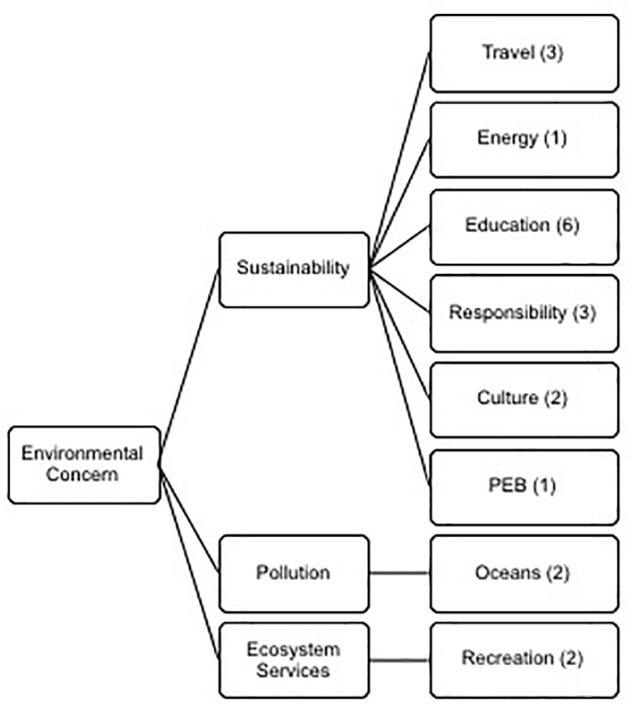
Thematic analysis of the meta-theme environmental concern displaying the three themes and eight sub-themes.

Marine social Scientist and surfer Easkey Britton, currently employed on the Horizon 2020 SOPHIE project, showed her research insights in her comment: “I think the real barrier or issue is even when we do access its’ the quality of the environment... I think we are at crisis point when it comes to the health of our oceans.” Three themes emerged under the major theme of environmental concern (See Figure 6): Sustainability (sub-themes: travel, energy, culture, pro-environmental behavior, education and responsibility), Pollution (sub-themes: oceans and plastics) and Ecosystem Services (sub-themes: recreation).

Our participants were well-placed to have an opinion on the challenge of the sustainability of their outdoor activities. None more so than Jim Kennedy, who as an adventure tourism provider described how he could see the beauty of it:

We are actually passionate, most people like us come into the business for the passion, of nature of our business, so that’s what’s happened, and we’re like-minded people who are pushing adventure tourism at a very high level, very professional level, but one of the benefits of that, we’re educating people, and sustainability, how to look after the sea, learn how to respect the waters, the walkways, the rivers, the trees, everything.

Sandra too was aware of the apparent contradictions in desiring to protect the wilderness and access it, sometimes by helicopter.

I’m definitely love being outdoors but I would definitely be hypocritical to say how much I care about it, I like to buy a lot of plane tickets I drive to a lot of rivers … it’s not something I feel 100% awesome about.

For Andreas in his post-athletic career role he reflects on sustainable travel:

It’s always a question when you travel so much. When I have teaching at University, it is one and a half hours away from where I live. It’s a lot of commuting and you always ask yourself if this is avoidable or not… I try to use the bike when I can.

He elaborates and states: “I think we are quite biased because here (e.g., Denmark) everything is about green energy and that’s the focus here and it’s not like this everywhere in the world.” He describes a range of pro-environmental behaviors (e.g., no plastic bag when shopping) and says how “with travel you see different cultures who have different attitudes toward garbage.” Cultural differences in attitudes toward sustainability have been shown in recent surveys. For example, in the European Commission (2017) only 56% of the Irish sample (*N* = 1,002) agreed that citizens themselves were not doing enough to protect the environment, compared to the higher level of awareness among respondents from Denmark (59%) and the EU (EU28: 66%). Not surprisingly, pro-environmental behavior including smart travel (e.g., public transport, walking, bike) are more commonly reported activities to reduce harmful emissions into the air in Denmark (41%) than Ireland (33%; EU28: 35%). Irish people rated air pollution as a lower priority issue (47%-lowest among EU 27) with similar discrepancies for items relating to the depletion of natural resources (EU-27 36%, IE 28%) and the loss of natural ecosystems (EU-27 26%; IE 19%). Among sport participants their values and attitudes may also be different depending upon their sport. Sandra gives the example of energy:

Kayakers are always super anti-hydro …cause dams ruin rivers but then the part of me that’s like has looked into different energy forms and stuff your like really hydroelectricity is quite a good renewable energy source compared to fossil fuels.

According to Tehillah McGuinness there is an imperative for those in outdoor sport to “*really push these things and get people involved…maybe not so much self-promotion but more taking care of the planet.”*

This concept of personal responsibility was clearly expressed by the former Pro-Surfer: “I think if we all take it upon ourselves I think hopefully that should make a difference… we have a huge responsibility to kind of to protect it and to encourage people.” The theme of education resonates with secondary school teacher Rosie Foley: “Why not start to educate from a very young age they want to know everything so we talked about plastic and the planet.” The participants insights reflected an understanding of the reciprocal benefits of human environment interactions. One of our surfers, Tehillah, sums this up succinctly: “the ocean is kind of what pays our bills in a way. We are earning money through being able to enjoy the ocean.” Our findings on environmental concern supports prior research which suggests that engaging with nature can have an impact on an individuals’ environmental concern (Ferrer-i-Carbonell and Gowdy, 2007; Brymer and Gray, 2010; Brymer and Schweitzer, 2013).

### Limitations

Qualitative sampling has limits with regard to generalizability and potential role of the researcher in biasing the research findings (Robson and McCartan, 2016). An uncontrolled factor in this qualitative study is the possibility that the sample participants were experiencing different stages of transition within their particular extreme sport career. A caveat is required in the interpretation of our findings which included three active participants and five who had retired from extreme sport. On the other hand, we had a broad sample drawn primarily from water sports with a gender balanced sample which is uncommon in studies of extreme sports (e.g., Frühauf et al., 2017). The findings are arguably more generalizable to the specific sports in question and this aligns with the view that high-risk sport participants should not be considered a homogenous group (Woodman et al., 2013). It is arguable that ultra-endurance sport activities in natural settings are distinct from traditional accounts of extreme sport activity and future research should address these distinctions. Similarly, ski-flying, the more extreme end of Nordic ski-jumping competition occurs an alpine setting whereas ski-jumping may be conducted in less natural settings. Regardless, the training settings are typically similar in their natural settings and attempts to categorize sport activities should include both competitive and training settings.

From a qualitative perspective (Robson and McCartan, 2016), researchers may question that interviewers work can be flawed by individual perceptions, inferring that recall biases can persist or that the researcher opinions can dictate the findings. This concern was addressed by employing a semi-structured approach to the interview which enabled flexibility in the discourse and the interview guide was largely informed by theories, thus the task was to confirm theory-based suppositions from an exploratory perspective.

### Pathways for Future Research

This exploratory study sows the seeds for future research which could employ a mixed-methods paradigm, and ideally apply a longitudinal perspective. Constructs such as “resilience” clearly resonated with the experience of this sample of extreme sport participants. Longitudinal research designs would enable the exploration of the dynamic emotional responses to nature interactions that may cultivate resilience.

A review of psychological recovery from an organizational psychology perspective suggested that natural environments seems particularly useful to replenish depleted resources (Sonnentag et al., 2017). In another achievement context, research is proliferating on the concept of recovery within elite sport (Kellmann and Beckmann, 2017; Frank et al., 2018). Developing interventions to help athletes engage with nature as a means of psychological recovery would be a worthy pursuit for researchers and practitioners. Thus we have two strands of research –one to examine how those in outdoor settings benefit from the challenge as well as the recovery processes enabled by the natural settings and another stream of research to explore how natural settings can facilitate recovery for athletes in both outdoor sports and indoor sports or those that occur in a hybrid or built environments (e.g., field games, or canoe slalom on artificial channels). One interesting question is whether athletes in outdoor sports, particularly those in extreme sport scenarios, gain more from recovery in natural settings than those with perhaps lower levels of nature connectedness (e.g., competitors in indoor racquet sports). These questions are worth of further qualitative exploration and quantitative analysis.

Recent research has focused on how Olympic athletes may benefit from greening of their competitive and training environments to enhance well-being (Donnelly et al., 2016). With the Olympic and Paralympic Games of Tokyo 2020 on the horizon and the third pillar of Olympianism being the “environment,” it may be useful to explore how athletes can become champions of a sustainable approach to ecosystem services.

The role of early childhood experiences highlighted by our interviewees provides a fitting backdrop for the development of both blue and green infrastructure to facilitate recreation and sport activity in natural spaces. The concept of Blueways, which are water and land-based routes with supporting infrastructure, may provide an opportunity for early access to sport activities and recreation in nature. Contemporary evidence supports both the health and mental health benefits of access to green space (Dadvand et al., 2014; Engemann et al., 2019). For example, a longitudinal study of 1 m Danish children reported a 55% lower risk of developing a mental disorder (after adjusting for other factors) if you grow up proximal to green spaces (Engemann et al., 2019).

Human-nature interactions cannot be simply reduced to concepts such as *exposure* (Frumkin et al., 2017), as nature has distinctive personal (e.g., nature relatedness; Mayer and Frantz, 2004; Nisbet et al., 2009), cultural and societal meaning. Meaning involves having aims and being absorbed by something that stimulates awe, curiosity, pleasure and other positively evaluated experiences (Howell et al., 2013). The increasing urbanization, environmental degradation in urbans settings and the digital immersion suggest that the meaning of nature should be preserved to benefit human and environmental health (Townsend et al., 2018). This role of meaning, place attachment and life orientation are worthy of concern for future research to ensure nature’s place is not displaced in our lives.

## Conclusion

Our sample displayed commonalities in their mindset, their connectivity with nature and attitudes toward the environment. Their differences paled in significance relative to their overlapping values, goals and responses to interacting with nature in the context of extreme sports. Interestingly, kayaker Sandra Hyslop said during her interview that “my dream, when I was about five or six, was to swim the Channel (e.g., English Channel)-still haven’t done it yet…one day!” From the English Channel to the top of Everest the nature of the challenge can change but the natural world still arouses a complex rich and consistent response among extreme sport performers.

## Ethics Statement

The project Title: 2016_11_20_ EHS GO Green Ex-Green Exercise in the Community: A Case Study Approach received ethical approval on Jan, 9th 2018 from the Education and Health Sciences Faculty REC at UL. Participants had the option to waive their right to anonymity so that the case -studies could include their identifiable personal narratives and scores from Principal Investigator: Tadhg MacIntyre. Other Investigators: Aoife Donnelly, Giles Warrington, Andree Walkin, Greig Oliver Collaborators Dr. Giovanna Caloguiri (INN Norway) & Prof. Marc Jones (Manchester Metropolitan University). This research study has ethical approval until December 2018.

## Author Contributions

TM, AW, and GO conducted the interviews and developed the *a priori* coding system. AW conducted the initial analysis with an independent analysis performed by TM. GC was the critical friend for the qualitative analyses. In addition to the aforementioned, SG, AD, JB, and GW were part of the research design, ethics application and contributed to both the original manuscripts and the revisions.

## Conflict of Interest Statement

The authors declare that the research was conducted in the absence of any commercial or financial relationships that could be construed as a potential conflict of interest.
