# Supplementary material for: An Exploratory Study of Extreme Sport Athletes’ Nature Interactions: From Well-Being to Pro-environmental Behavior
Source: Front Psychol. 2019 May 28;10:1233. doi: 10.3389/fpsyg.2019.01233 (PMC6546823; doi:10.3389/fpsyg.2019.01233)
Supplement: Supplementary file 2 [file Data_Sheet_1.PDF]

Semi-Structured Interview Protocol

1. Let us start by you telling me about your background and experience in outdoor exercising and sport. *(early childhood exposure to nature)*

2. Can you tell me about your career and sporting achievements, personal milestones and most memorable moments? *(how meaningful are they to outdoor nature related experiences)*
3. Has there been any particular person/people who have influenced you? *(Parenting, role models, mentors and anti-mentors)*
4. Have you faced any adversities in sport or major life events which you might like to discuss? How has getting into nature helped you cope? *(Do you seek nature for therapeutic purposes –mental health, to feel better –well-being, to be with others-social support, for challenge-to build resilience)*
5. How did you cope and thrive in response to the adversities you have had faced?
6. What would you say are your daily stressors are how do you cope with these? Does nature help?
7. What activities do you do outdoors? How frequently do you do these activities, how effortful? What duration? Do you do these sports with others or alone? *(FITT of activity in Nature - is it different to gym/indoor/pitch training)*
8. Do you have a favourite natural space? Why do you go there? (Is the purpose Restoration, Energising, Creativity? Innovation? Life Decisions?)
9. Do you associate certain places with certain emotions? Can you give an example? (Restorative space, therapeutic landscape, place blindness-somewhere local that you didn't go as a kid but go now)
10. How do you find being physically active in nature affects your mood, *(well-being, state of mind, creativity, empathy and social interactions)*
11. *Are there any other barriers or risks to being active in nature? (injury)* In what way if any does the weather affect you and your physical activity? *(Seasonal variation)*
12. What are your thoughts on using nature to reduce barriers to looking after our mental health? (*Is there stigma? Does nature offer a low-stigma approach)*
13. What are your views on sustainability and the environment? *(How can contact with nature change our attitudes towards the environment, recycling, active travel)*
14. Would you say you are connected with nature, in particular GREEN NATURE?
15. If you could change one thing to increase access to blue/green spaces what would it be? (green/blue infrastructure)
16. How and why would you recommend people get involved in blue or green exercise? (Youth? Elderly? Athletes? Communities? Citizen science)
17. Could you show me your phone screen image? Can you tell me about it? (Technological nature-to take your phone or not take your phone?) Does it change your emotions? Do you need to be in nature for it to impact upon you?

Closure

18. Anything else you would like to add on blue spaces or how water environments can benefit health? Conclusion-Tell them what will happen next…

Probes and follow up questions

- Is there anyone who influenced you to choose that?
- Can you elaborate on that point? Can you give me an example?
- If the opposite was the case how would you feel, react?
- Is that the case in other situations and circumstances? Such as …
- What was I that swayed you to do that?
- What is it you mean by [term or phrase]?
- When you say, [term or phrase], what are you actually doing?
- It sounds like you are saying, ". . . .". Is that a fair summary?
- How do you do that?
- How did others respond to that?
- If I were watching you do this, what would I see?

- Why does that stand out in your memory?
- Why do you think you noticed that?
- *Keep on track*
- I'd like to understand more about how this relates to the earlier topic we were talking about.
- Can you recall the associations that lead you from our original topic to this one?
- *Emotions*
- Can you say something about why this issue generated such emotion?
- What aspects of this do you think prompted these emotions?
- Why was it that this affected your mood?
- *Emotion and mood words*
- Feeling, sentiment, sensation, experience, disposition, attitude, temperament, humour, state of mind, frame of mind
