# Supplementary material for: An Exploratory Study of Extreme Sport Athletes’ Nature Interactions: From Well-Being to Pro-environmental Behavior
Source: Front Psychol. 2019 May 28;10:1233. doi: 10.3389/fpsyg.2019.01233 (PMC6546823; doi:10.3389/fpsyg.2019.01233)
Supplement: Supplementary file 3 [file Data_Sheet_2.PDF]

Apriori Coding Scheme

1. **Early Childhood**
   -Exposure in blue green nature
   -Blue Green play
   -Positive Experiences/strong memories

2. **Youth Sport**
   -Specialisation
   -Multi Sport
   -Outdoor Sport
   -Achievements/Special moments

3. **Role models**
   -Positive/Negative people
   -Parental influence
   -Sibling Rivalry
   -Sports People

4. **Sport**
   - Specialisation
   - Extreme
   -Technique
   -Recreation/Fun
   -Multi Sport
   -Outdoor Sport
   -Achievements/Special moments

5. **Stress**
   -Nature for coping
   -Daily hassles
   -Life Stress
   -Resilience
   -Post Trauma growth

6. **Challenge of outdoors**
   -Immersion
   -Risk
   -Solo
   -Effortful
   -Resilience
   -Memorable
   -Confidence
   -Excitement
   -Culture

7. **Stigma**
   -Nature as Low/high stigma

8. **Restorative Space**
   -Emotional Spaces
   -Place Blindness
   -Connectedness
   -Favourite Natural Space

9. **Emotional response to Nature**
   -Viewing v Immersed
   -Engagement idea

10. **Access to Nature**
    -Contact
    -Barriers
    -Facilitators
    -Carbon Footprint

11. **Environmental concern**
    -Positive Negative
    -Active Travel
    -Recycling
    -Sharing Nature

12. **Education & Awareness**
